# Plasma sterols and vitamin D are correlates and predictors of ozone-induced inflammation in the lung: A pilot study

**DOI:** 10.1371/journal.pone.0285721

**Published:** 2023-05-15

**Authors:** Alexia N. Perryman, Hye-Young H. Kim, Alexis Payton, Julia E. Rager, Erin E. McNell, Meghan E. Rebuli, Heather Wells, Martha Almond, Jamie Antinori, Neil E. Alexis, Ned A. Porter, Ilona Jaspers

**Affiliations:** 1 Curriculum in Toxicology and Environmental Medicine, University of North Carolina at Chapel Hill, Chapel Hill, NC, United States of America; 2 Center for Environmental Medicine, Asthma, and Lung Biology, University of North Carolina at Chapel Hill, Chapel Hill, NC, United States of America; 3 Department of Chemistry, Vanderbilt University, Nashville, TN, United States of America; 4 Department of Environmental Sciences and Engineering, Gillings School of Global Public Health, University of North Carolina at Chapel Hill, Chapel Hill, NC, United States of America; 5 Department of Pediatrics, University of North Carolina at Chapel Hill, Chapel Hill, NC, United States of America; Endocrinology and Metabolism Population Sciences Institute, Tehran University of Medical Sciences, ISLAMIC REPUBLIC OF IRAN

## Abstract

**Background:**

Ozone (O_3_) exposure causes respiratory effects including lung function decrements, increased lung permeability, and airway inflammation. Additionally, baseline metabolic state can predispose individuals to adverse health effects from O_3_. For this reason, we conducted an exploratory study to examine the effect of O_3_ exposure on derivatives of cholesterol biosynthesis: sterols, oxysterols, and secosteroid (25-hydroxyvitamin D) not only in the lung, but also in circulation.

**Methods:**

We obtained plasma and induced sputum samples from non-asthmatic (n = 12) and asthmatic (n = 12) adult volunteers 6 hours following exposure to 0.4ppm O_3_ for 2 hours. We quantified the concentrations of 24 cholesterol precursors and derivatives by UPLC-MS and 30 cytokines by ELISA. We use computational analyses including machine learning to determine whether baseline plasma sterols are predictive of O_3_ responsiveness.

**Results:**

We observed an overall decrease in the concentration of cholesterol precursors and derivatives (e.g. 27-hydroxycholesterol) and an increase in concentration of autooxidation products (e.g. secosterol-B) in sputum samples. In plasma, we saw a significant increase in the concentration of secosterol-B after O_3_ exposure. Machine learning algorithms showed that plasma cholesterol was a top predictor of O_3_ responder status based on decrease in FEV1 (>5%). Further, 25-hydroxyvitamin D was positively associated with lung function in non-asthmatic subjects and with sputum uteroglobin, whereas it was inversely associated with sputum myeloperoxidase and neutrophil counts.

**Conclusion:**

This study highlights alterations in sterol metabolites in the airway and circulation as potential contributors to systemic health outcomes and predictors of pulmonary and inflammatory responsiveness following O_3_ exposure.

## Introduction

Inhalation exposure to ground-level ozone (O_3_) produces several respiratory symptoms including increased airway hyperresponsiveness, decreased lung function, and lung inflammation [1–3]. However, O_3_-induced health effects are not restricted to the lung as O_3_ is associated with increased cardiovascular morbidity and mortality, diabetes, and neurodegenerative diseases [4–9]. Yet, it is unclear how inhalation exposure to O_3_ promotes development of these adverse cardiometabolic and systemic health outcomes.

Numerous factors such as genetics, age, sex, existing respiratory disease, and dietary deficiencies have been identified to modify susceptibility to O_3_-induced health effects [9–13]. Interestingly, baseline metabolic state also impacts susceptibility to O_3_. For example, obesity is associated with airway hyperresponsiveness and enhanced inflammatory responses in rodents and greater lung function decrements in humans in response to O_3_ [14–16], which may be due in part to altered lipid phenotype in the lung [17]. Additional work in rodent models fed a high cholesterol diet show higher baseline levels of lung inflammation and enhanced proinflammatory responses to O_3_ [18,19]. Taken together, these studies highlight metabolic dysregulation as both an emerging endpoint of interest and a susceptibility factor to explain O_3_-induced health effects within and outside the lung.

Ongoing studies are elucidating the factors that elicit systemic response to O_3_. O_3_ increases circulating stress hormones that contribute to lung inflammation through a centrally mediated activation of the stress response pathway, the hypothalamus-pituitary-adrenal axis [20–26]. Transcriptomic studies of O_3_-exposed rats show gene expression changes in the lung and several organs consistent with glucocorticoid activation [22,24,27]. Given that O_3_ alters systemic lipid metabolism provides impetus to evaluate the breadth of lipid alterations not only in the lung, but also in circulation following exposure.

Lipidomic and metabolomic studies have primarily identified changes in arachidonic acid metabolites and polyunsaturated fatty acids following O_3_ exposure [23,26,28,29]. However, recent transcriptomic studies in rodents have identified upregulation of cholesterol biosynthesis pathway genes in the lung following O_3_ exposure[30–33]. Cholesterol is the major neutral lipid component of airway surface liquid and important precursor to hormones demonstrated to modulate O_3_ responses (i.e. corticosteroids and sex steroids). For this exploratory study, we hypothesized that exposure to O_3_ is associated with changes in sterol and oxysterol lipid profiles in the lung and circulation and systemic changes in lipid mediators such as oxysterols may be associated with known measures of O_3_-responsiveness (e.g., neutrophilia, lung function decrements). Using targeted Liquid Chromatography Mass Spectrometry (LC-MS), we measured 24 sterol and oxysterol species in induced sputum and plasma samples from healthy and asthmatic adult volunteers before and 6 hours after exposure to 0.4 parts per million (ppm) for 2 hours. Pulmonary function, sputum differential cell counts, and soluble markers of inflammation in blood and sputum were assessed at baseline and following exposure to O_3_. We found that O_3_ exposure is associated with decrease in sterol profile in the airways and significant increase in oxysterols in circulation. At baseline, asthmatics and non-asthmatics had differing sterol profiles. Elevated plasma cholesterol and lower vitamin D status was associated with lung function response and disease status following O_3_ exposure. Specifically, baseline vitamin D status was positively associated with lung function and inversely associated with neutrophilia after exposure in non-asthmatics. Findings from this study collectively support the need to examine lipid dysregulation as both a potential determinant and health outcome of O_3_ response.

## Materials and methods

### Study volunteer recruitment

Healthy adult non-smoking volunteers with less than one-half pack year history between the ages of 18–40 years old were recruited as part of prior clinical study (NCT00840528) and asthmatics were recruited for clinical study (NCT00287365). Written informed consent was obtained at the time of the clinical studies. Additional findings have been previously published for these cohorts [10,34–42]. All studies were performed in accordance with the Declaration of Helsinki with protocols approved by the Institutional Review Board at the University of North Carolina at Chapel Hill.

### In vivo O_3_ exposures

A baseline visit was conducted for each of the volunteers to measure lung function and collect blood. On a subsequent visit (no more than 5 months later), lung function tests were performed then individuals were exposed to 0.4ppm O_3_ for 2 hours with intermittent light exercise for 15 minutes as described previously [10]. At 6 hours post-exposure, lung function tests, blood collection, and sputum induction were performed. For all asthmatic volunteers, albuterol was administered prior to sputum induction. Blood samples were collected in sodium citrate tubes for isolation of plasma. Sputum samples were analyzed for total cell count and cell differentials and further processed to obtain cell-free supernatants. Both plasma and cell-free sputum supernatants were stored at -80°C prior to downstream analysis of sterol derivatives and cytokines by UPLC-MS and ELISA, respectively, as detailed in the supplemental methods.

### Missing data

A single baseline sputum sample from one male asthmatic subject was not available for cytokine analysis and insufficient volume for myeloperoxidase (MPO) quantification occurred in 4 samples, otherwise all baseline and post-exposure plasma, sputum and lung function measurements were available and included in mixed model analyses.

### Statistical analyses

The lower limit of detection was calculated as 2.5 standard deviations above the average signal of the blank for each analyte measured by multiplex ELISA by fitting a 4-parametric logistic curve function with weight in GraphPad Prism (v.9). Analytes detected in less than 25% of samples for a given sample type (sputum or plasma) were excluded from subsequent individual analyses described in the supplemental methods. Individual analytes were screened for interaction between disease status, sex, and O_3_ response using a 3-way mixed-effects model with repeated measures with Bonferroni correction for multiple comparison within each analyte. In the absence of significant 3-way interaction, subsequent confirmatory analyses of two-way interactions or simple main effects of individual factors by two-way mixed-effects model (*Sex x* O_3_ or *Disease x O*_*3*_) with repeated measures or paired Wilcoxon rank sign test were respectively performed. The resulting p-values from the ANOVAs and post hoc testing were adjusted using Benjamini Hochberg method [43] All demographic information, lung function, sputum characteristics, cytokine data, and sterol data are summarized in S4–S7 Tables.

### Data organization and processing for machine learning

For generation of machine learning models, all data were processed, and subsequent analyses carried out in R software (v 4.1.2). Similar to the individual variable analyses, background filters were initially implemented to remove cytokine, cell differential, and sterol measures that were universally lowly expressed and subjects with less than 25% of observed data. Next, data imputation was carried out on the remaining missing data points using either Quantile Regression Imputation of Left-Censored data (QRILC) using the imputeLCMD package [44] for all variables except lung function, which was imputed for missing data using random forest modeling through the missForest package [45]. The supplemental methods further detail the background filters and reasoning for performing imputation model selection.

### Supervised machine learning models to predict inflammatory and lung function response

Supervised machine learning models were used to evaluate the degree to which sterol metabolites and subject demographic data would be able to predict inflammatory or lung response to O_3_ exposure in human volunteers. Additional information regarding the predictors and covariates incorporated in the models are discussed in the supplemental methods. Outcome variables, inflammatory response and lung function response, were dichotomized into two classes: non-responders and responders using previously published cut off values [46,47]. Inflammatory responders were defined having at least a 10% increase in neutrophil percentage following O_3_ exposure [46]. Of the 24 subjects included, this resulted in 8 being classified as non-responders and 16 as responders. Lung responders were defined as having at least a 5% decrease in percent predicted forced expiratory volume in 1 second (FEV1) following O_3_ exposure [47].

Three algorithms were evaluated to first identify which model could best predict the evaluated outcomes, which included random forest (RF), support vector machine (SVM), and k nearest neighbor (KNN) chosen due to their acceptance within this field of research and published utility towards predicting toxicological outcomes using molecular data [48,49]. Each of their mechanisms and tuning parameters are expanded upon in the supplemental methods. The performance of these three models were compared, using sets of predictor variables spanning only the molecular mediators, as well as predictor variables spanning the molecular mediators and subject demographic data, to identify which model(s) could predict inflammatory and lung response outcomes the best.

### Evaluation of model performance and interpreting best performing machine learning model

Model performance was assessed using confusion matrix metrics, which quantify an algorithm’s ability to classify subjects into the correct class. Area under the curve [AUC of the receiver operating characteristic (ROC) curve] was calculated as another metric to assess model performance, quantifying measure performance at varying classification thresholds as described previously [49]. To increase the generalizability of the models to data it had not “seen” before, 5-fold cross-validation was performed and confusion matrix and AUC were averaged across the 5 iterations as described in the supplemental methods.

A decision boundary plot was generated to visualize how the best performing model would separate the two responder classes based on varying concentrations of two sterol metabolites more routinely measured in clinical studies, cholesterol and 25-hydroxyvitamin D. This could potentially elucidate potential biological significance between those two features (biomarkers) and inflammatory or lung response.

### Sterol correlation analysis

Spearman’s rank correlation analysis was performed to assess associations between sterol precursor concentrations in plasma and O_3_ induced lung outcomes. This was done in R (v 3.6.2) using the psych package [50] and then all correlations with were visualized using the corrplot package [51].

## Results

### Subject demographics

We analyzed plasma and sputum from a total of 12 healthy and 12 asthmatic human volunteers. Our study included both males (N = 6) and females (N = 6) for each group. Accordingly, we have summarized race, age, body mass index (BMI), sputum cell characteristics, cytokines, and sterols for all subjects and included data stratified by sample type and sex (S1–S7 Tables).

### O_3_ alters sterol profiles within the lung

**Fig 1:**
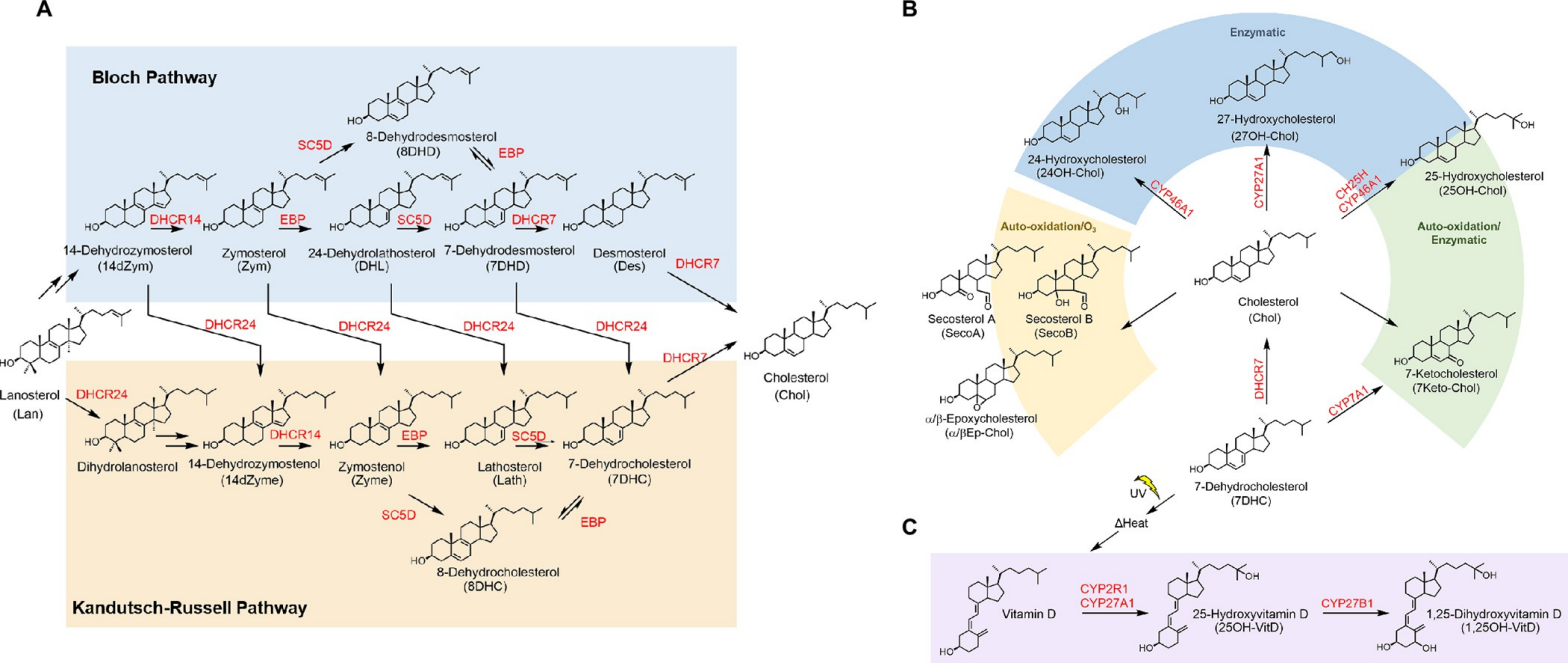
A) Post-lanosterol cholesterol biosynthesis pathway proceeds through two branches, Bloch (blue) or Kandutsch-Russell Pathway (orange). B) Formation of oxysterols from cholesterol occurs through enzymatic (blue), autooxidation (yellow), or both enzymatic and autooxidation (green) routes. C) Vitamin D is a secosteroid prohormone formed conversion of 7-dehydrocholesterol (7-DHC), a cholesterol precursor, by UV radiation in the skin. Vitamin D undergoes subsequent hydroxylation reactions to form 25-hydroxyvitamin D (25OH-VitD) and the bioactive metabolite, 1,25-hydroxyvitamin D.

**Fig 2:**
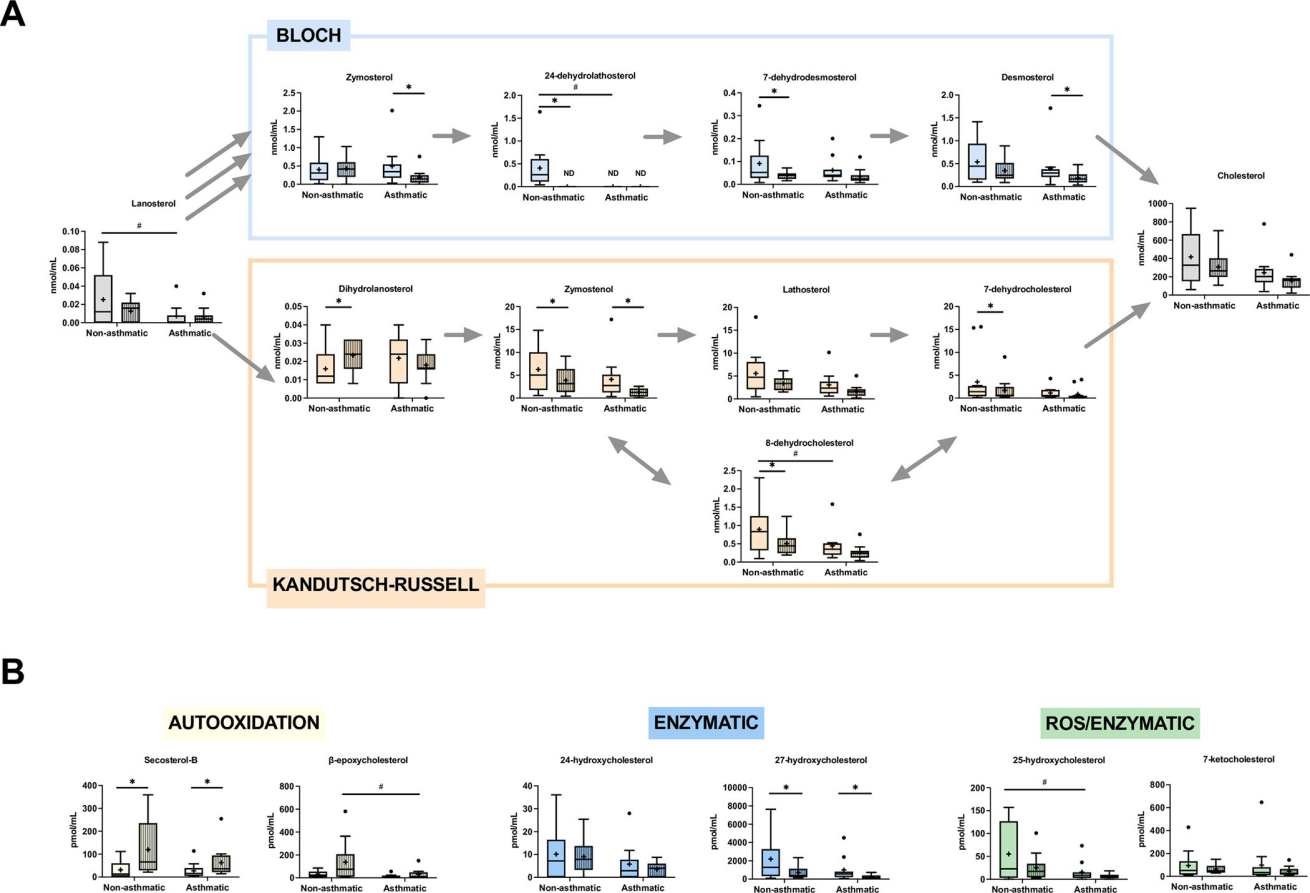
Sterols and oxysterols in sputum before and after ozone exposure in asthmatics and non-asthmatics. A) Sterols from both arms of the cholesterol biosynthesis pathway (Bloch-Blue, Kandutsch-Russell-Orange) were measured in sputum. B) Oxysterols generated enzymatically (blue), enzymatically and by cholesterol autooxidation (green), or cholesterol autooxidation (yellow) were quantified by UPLC MS. For box and whiskers plot, line represents the median, ‘+’ represents the mean. Baseline and post-exposure (vertical stripes) time points were compared by Wilcoxon matched-pairs signed rank test. *p<0.05, **p<0.01, ***p<0.001, N = 12. Differences between asthmatics and non-asthmatics were compared by mixed-effects model with repeated measures. #-p<0.05, ##-p<0.01, ###-p<0.001, N = 12.

We performed targeted analysis of sterol, oxysterol, and secosteroid species in human plasma and sputum samples. Both the Bloch and Russell-Kandutsch pathways are differentially used for the final steps of cholesterol synthesis in a cell-specific manner (Fig 1A) [52,53]. There were higher concentrations of cholesterol precursors generated through the Kandutsch-Russell pathway in sputum samples from both non-asthmatics and asthmatics (Fig 2A, S6 Table). Additionally, several oxysterols are generated from cholesterol via autooxidation or enzymatic reactions (Fig 1B and 1C).

O_3_ exposure was associated with an increase in SecoB and decrease in 27-hydroxycholesterol in the sputum from both non-asthmatics and asthmatics (Fig 2, S6 Table). O_3_ exposure was associated with a decrease in several cholesterol precursors. For asthmatics, zymosterol, desmosterol, and zymostenol were significantly decreased (Fig 2A, S6 Table). For non-asthmatics, 7-dehydrocholesterol, 8-dehydrocholesterol, and 7-dehydrodesmosterol were significantly decreased (Fig 2A, S6 Table). In contrast 24-dehydrolathosterol and dihydrolanosterol, were significantly increased in the sputum from non-asthmatics (Fig 2A, S6 Table).

### O_3_-induced changes in systemic sterol and oxysterol levels

**Fig 3:**
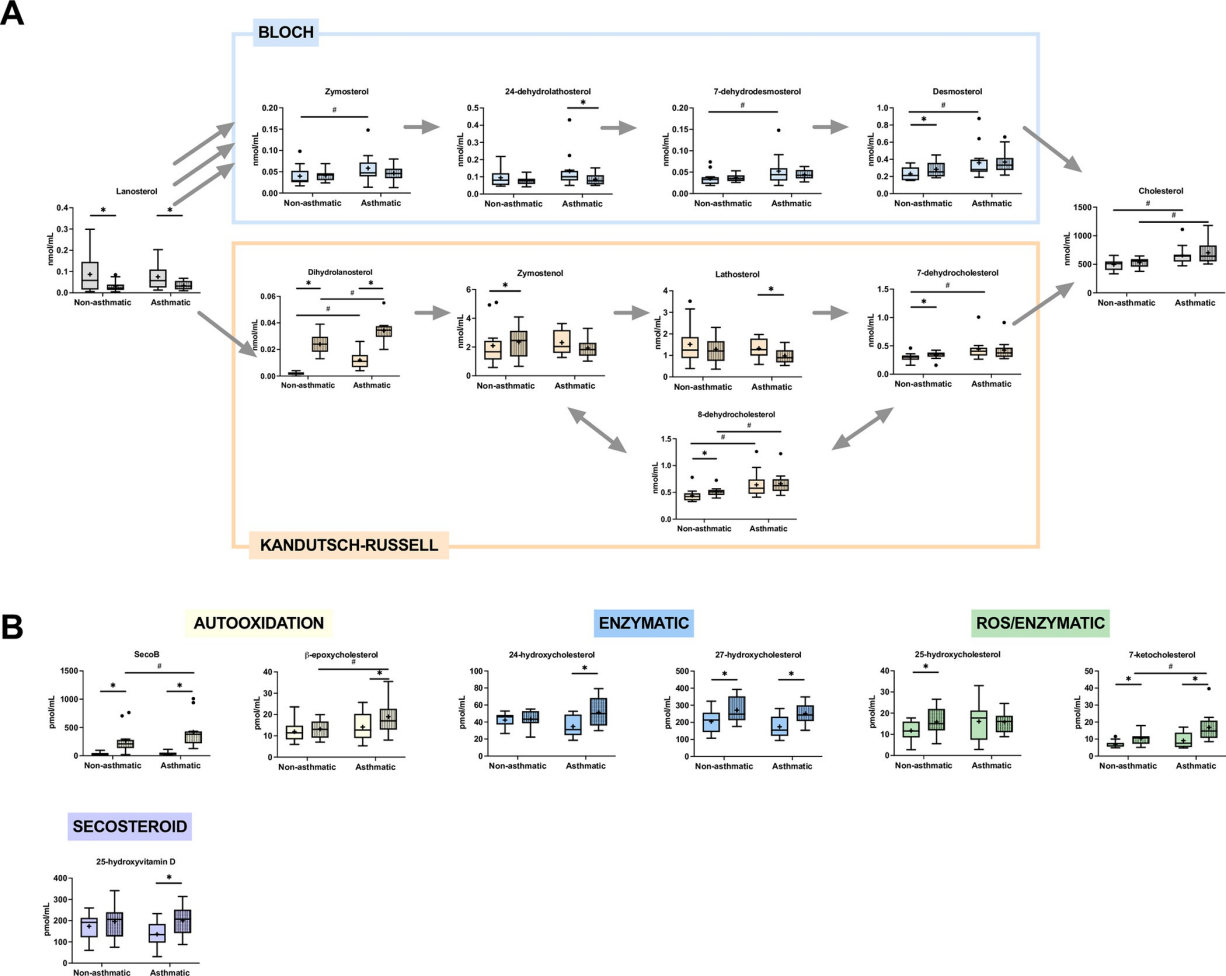
Sterols, oxysterols, and 25-hydroxyvitamin D levels in plasma before and after ozone exposure in asthmatics and non-asthmatics. A) Sterols from both arms of the cholesterol biosynthesis pathway (Bloch-Blue, Kandutsch-Russell-Orange) were measured in plasma. B) Oxysterols generated enzymatically (blue), enzymatically and by cholesterol autooxidation (green), or cholesterol autooxidation (yellow) were quantified alongside 25-hydroxyvitamin D (purple) by UPLC MS. For box and whiskers plot, line represents the median, ‘+’ represents the mean. Baseline and post-exposure (vertical stripes) time points were compared by Wilcoxon matched-pairs signed rank test. *p<0.05, **p<0.01, ***p<0.001, N = 12. Differences between asthmatics and non-asthmatics were compared by mixed-effects model with repeated measures. #-p<0.05, ##-p<0.01, ###-p<0.001, N = 12.

To evaluate if O_3_ exposure is associated with systemic changes in sterol concentrations, we also analyzed concentrations of cholesterol precursors and derivatives in subject-matched plasma samples. Like the sputum samples, there were higher concentrations of cholesterol precursors in the Kandutsch-Russell pathway than in the Bloch pathway (Fig 3A). However, the direction of changes in sterols were more mixed in comparison to those observed in sputum. Several of these changes were shared between non-asthmatics and asthmatics. Specifically, there was a significant increase in 27-hydroxycholesterol, 7-ketocholesterol, and 24-dehydrolathosterol following O_3_ exposure. Most notably, O_3_ exposure was associated with a significant increase in plasma secosterol-B (SecoB) in both groups (Fig 3B, S7 Table). Though SecoB was detected in both plasma and sputum, the isomer SecoA was not detectable in either plasma or sputum samples for any participant. In contrast, plasma concentrations of lanosterol were decreased following exposure in both groups (Fig 3A, S7 Table) I.

Unique to non-asthmatics were significant increases in 7-dehydrocholesterol, 8-dehydrocholesterol, desmosterol, and 25-hydroxycholesterol (Fig 3A, S7 Table). In asthmatics, lathosterol and 24-dehydrolathosterol were decreased (Fig 3A, S7 Table). In contrast, dihydrolanosterol, enzymatically-derived oxysterols (24-hydroxycholesterol, 27-hydroxycholesterol), oxidation-derived (7-ketocholesterol and β-epoxycholesterol), and secosteroid (25-hydroxyvitamin D) all significantly increased following O_3_ exposure (Fig 3, S7 Table).

### Baseline differences in sterols

We assessed whether sterol and oxysterol concentrations differed between asthmatics and non-asthmatics. At baseline, asthmatics had lower sputum 25-hydroxycholesterol, lanosterol, 8-dehydrocholesterol, and 24-dehydrolathosterol (Fig 2, S6 Table). Conversely, asthmatics had higher plasma 7-dehydrocholesterol, 8-dehydrocholesterol, dihydrolanosterol, zymosterol, 7-dehydrodesmosterol, desmosterol, and cholesterol (Fig 3, S7 Table). Interestingly, 24-dehydrolathosterol was only detectable in non-asthmatics at baseline. At the post-exposure timepoint, β-epoxycholesterol was significantly lower in asthmatics compared to non-asthmatics in sputum samples (Fig 2B, S6 Table). In plasma, SecoB, 7-ketocholesterol, β-expoxycholesterol, dihydrolanosterol, 8-dehydrocholesterol, and cholesterol were all significantly higher in asthmatics than non-asthmatics (Fig 3, S7 Table).

### Machine learning model performance comparison of O_3_ responder status

O_3_ responder status was based on previously reported criteria for a robust pro-inflammatory O_3_ response based on a >10% increase in post vs pre-exposure (%Neutrophils Post—% Neutrophils Pre) [54]. To determine if plasma sterol concentrations could be utilized for predicting O_3_ responsiveness, machine learning models were constructed using all subjects. When predicting inflammatory response, SVM prediction with a polynomial kernel was the best performing model predicting modestly well (AUC = 0.57). Model performance of radial SVM remained the same with and without inclusion of covariates (S8 Table). Inclusion of covariates led to a decrease in predictivity for linear SVM, RF, and KNN and remained the same for polynomial SVM. Overall, most of these models did a decent job of classifying responders (sensitivity ≥ 0.68) but struggled to correctly label non-responders (specificity ≤ 0.30) (S9 Table).

When predicting lung response, SVM prediction with a radial kernel was the best performing model predicting very well (AUC = 0.93). Model performance of radial SVM remained the same with and without inclusion of covariates (S8 Table). After radial SVM, models in descending order of predictivity included RF, KNN, linear SVM, and polynomial SVM. Generally, most algorithms were able to correctly label responders and non-responders to a high degree (sensitivity ≥ 0.63 and specificity ≥ 0.73, respectively) (S9 Table). Overall, models were substantially better at predicting lung response than inflammatory response, likely attributable to the unbalanced inflammatory response variable with 8 non-responders and 16 responders.

**Fig 4:**
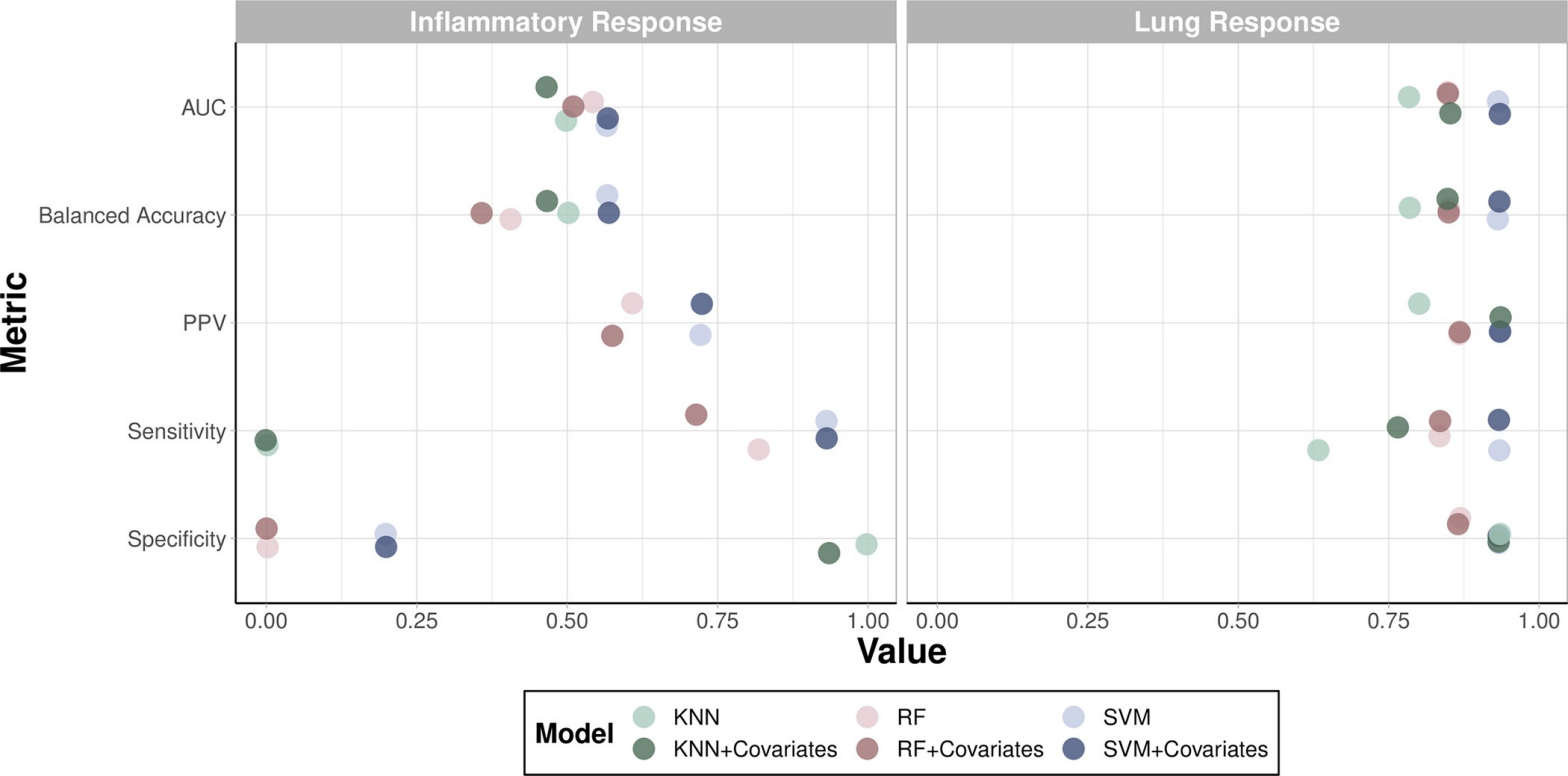
Confusion matrix for predictive models for inflammatory or lung response to O_3_ using baseline plasma sterol concentrations. The following machine learning models were run with (opaque dots) and without (transparent dots) covariates (sex, BMI, ethnicity, visit season, age, race): random forest (RF)—red, support vector machine (SVM)—blue, and k nearest neighbor (KNN)—green. The area under the curve (AUC) of the receiver operating characteristic (ROC) curve, balanced accuracy, positive predictive value (PPV), sensitivity, and specificity of each model are displayed.

Confusion matrix metrics for both lung and inflammatory response variables are visualized for RF, KNN, and linear SVM models with and without covariates (Fig 4). When interpreting the best performing RF model for predicting inflammatory response (i.e., RF with covariates included), 12 out of 19 sterol predictors fell above random noise (S10 Table). Out of these, SecoB was the top predictor. When interpreting the best performing model for predicting lung response (i.e., RF with covariates included), 8 out of 19 predictors fell above random noise. As the top predictor, cholesterol emerged as a major driver for lung response prediction with it being twice as important as the closest predictor (S11 Table).

### Visualization of best performing model and contributions from cholesterol and vitamin D

**Fig 5:**
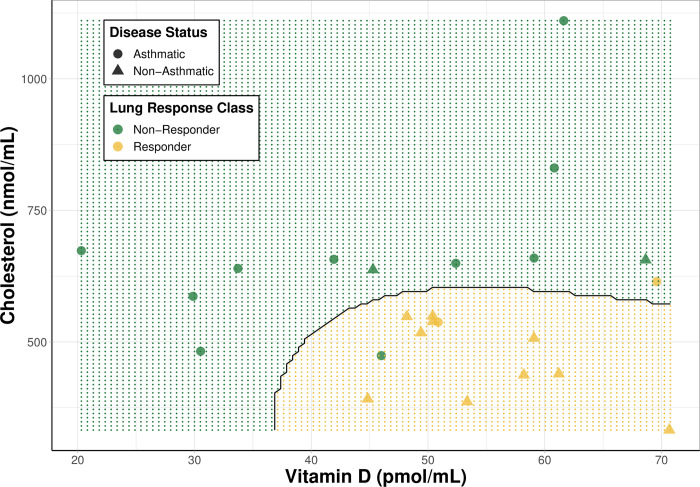
Decision boundary plot for SVM model predicting lung response class. Cholesterol and 25-hydroxyvitamin D were used as predictors visualizing responder status [non-responders(green) and responders (yellow)] and disease status [non-asthmatics (triangles) and asthmatics (circles)]. The shaded regions are the model’s prediction of a subject’s lung response class at a given cholesterol and 25-hydroxyvitamin D concentration.

While we observed significant changes in several sterol species in association with asthma status or O_3_ exposure, most of these sterols are not routinely measured in clinical settings. For this reason, a decision boundary plot was used to visualize the best performing model, radial SVM for lung response prediction, this time only using two predictors: cholesterol, the top predictor, and 25-hydroxyvitamin D, a systemic marker often measured during routine blood panels. Disease status was added to the visualization after prediction to determine if it coincided with clustering of non-responders and responders and disease status coincided with those clusters (Fig 5). Subjects with robust lung function responses to the O_3_ exposure were predominantly non-asthmatics with lower cholesterol and higher plasma 25-hydroxyvitamin D concentrations.

### Baseline plasma vitamin D status is associated with O_3_-induced changes in lung function and pulmonary mediators in non-asthmatics

**Fig 6:**
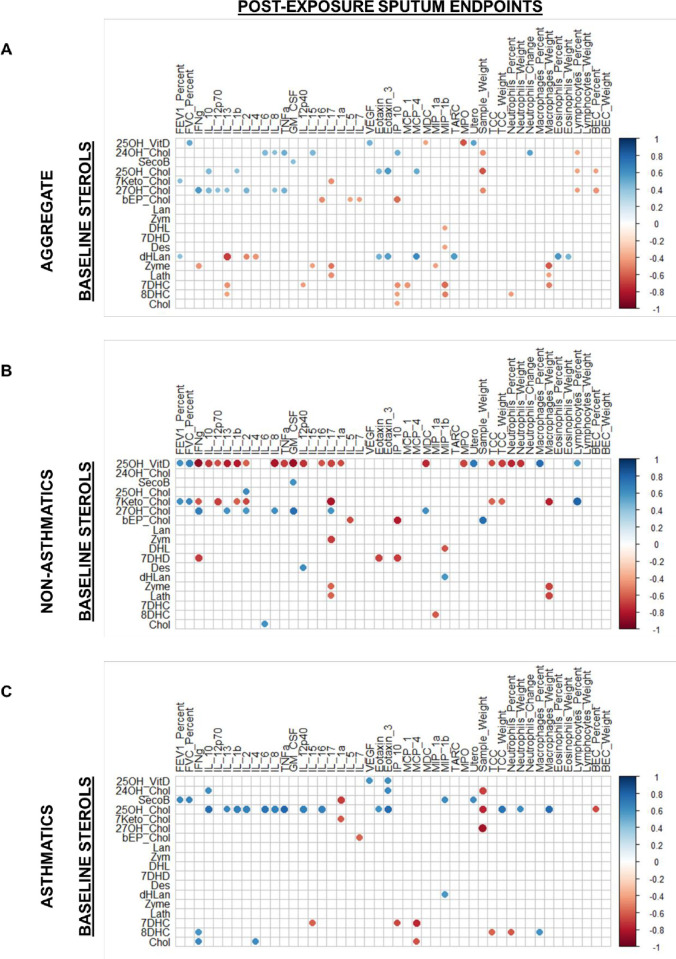
Spearman’s rank correlation for baseline plasma sterols and oxysterols with post-O_3_ exposure sputum endpoints. Correlations were stratified based on disease status for A) all subjects, B) non-asthmatics, and C) asthmatics. Only significant correlations (p<0.05) are shown.

Of particular interest for this study was the identification of association between systemic levels of sterol precursors/derivatives and lung outcomes post-exposure to O_3_: lung function (FEV1, FVC), immune cell counts, cytokines, and soluble inflammatory mediators. Given the unequal distribution of lung function responders between asthmatics (N = 2) and non-asthmatics (N = 10), we analyzed correlations between plasma sterols and lung endpoints after O_3_ exposure separately (Fig 6). Through this analysis, we observed positive correlation between plasma 7-ketocholesterol and lung function measures and negative correlation between 7-ketocholesterol and percent macrophages and in the sputum after O_3_ exposure (Fig 6A). In asthmatics, SecoB was associated with cytokines interleukin (IL)-4, IL-8, and IL-13 in sputum after exposure. Also, plasma zymosterol concentration was inversely associated with sputum TARC concentration. Interestingly, we found baseline plasma 25-hydroxyvitamin D levels to be correlated with several endpoints of interest in non-asthmatics (Fig 6A). Specifically, plasma 25-hydroxyvitamin D was positively associated with lung function measures (FEV1 and FVC) and uteroglobin in sputum. 25-hydroxyvitamin D was negatively associated with neutrophil and MPO in sputum. In other words, low plasma 25-hydroxyvitamin D levels were associated with higher neutrophilia and MPO levels in sputum and lower lung function after O_3_ exposure.

## Discussion

Recent climate change prediction modeling estimate that in the U.S. warming temperatures will be associated with higher O_3_ levels in some regions [55]. Hence, O_3_ continues to present a growing threat to public health. Of emerging importance is the contribution of metabolic predisposition to O_3_-induced adverse health effects. We conducted an exploratory study profiling several sterol precursors and derivatives to assess their association with well-studied endpoints of O_3_-responsiveness in healthy and asthmatic human volunteers. Our data show that circulating sterols are modified after O_3_ exposure and Vitamin D levels may be predictive of O_3_ responsiveness.

O_3_ can directly oxidize the unsaturated bond between the 5^th^ and 6^th^ carbons in cholesterol to form these oxysterols. Based on our previous studies and existing literature, oxysterols, such as SecoA and β-epoxycholesterol are significantly elevated in bronchoalveolar lavage fluid collected from humans and lung homogenates of rats exposed to O_3_ [56–58]. We observed an increase in SecoB in sputum samples in line with past findings (Fig 2B). By extending our analysis to plasma samples, we also observed a significant increase in plasma SecoB concentrations in both asthmatics and non-asthmatics (Fig 3B). Despite modest inflammatory response predictivity, SecoB still emerged as the top predictor in RF models built to leverage cytokine and sterol distributions to predict inflammatory responder status (S10 Table). Notably, both SecoA and SecoB have been identified in numerous extrapulmonary tissues such as plasma, brain, and liver [59,60]. It is possible that SecoA and SecoB are generated in the lung as a direct reaction of O_3_ and cholesterol are leaked into circulation due to increased pulmonary permeability from either exercise or O_3_ exposure. Although SecoA and SecoB are commonly referred to as O_3_-derived oxysterols, both can be produced through autooxidation of cholesterol by O_3_-independent mechanisms (49–51). One such pathway is neutrophilic inflammation in which myeloperoxidase (MPO), an enzyme primarily found in granulocytes, generates reactive oxygen species [60–62]. Given that the peak in peripheral neutrophilia occurs 4–6 hours post-exposure [63,64], it is possible that the generation of reactive oxygen species (ROS) by neutrophilic MPO may contribute to the increased levels of the SecoB in plasma following exposure to O_3_.

Regardless of the source or synthesis pathway, this is the first report demonstrating significant increases in systemic SecoB following O_3_ exposures. Long-term O_3_ exposure is associated with cardiovascular morbidities development [4–8]. SecoA and SecoB, have been identified in human atherosclerotic plaques and demonstrated to have proatherogenic properties such as promotion of macrophage recruitment and adhesion, induction of endoplasmic reticulum stress, and apoptosis in human umbilical vascular endothelial cells [61,65,66]. In human plasma samples, concentrations of SecoB were 70-1690nM in 6 out of 8 patients with advanced carotid disease while in healthy controls levels measured above the limit of detection were only present 1 of 15 patients [61,67,68]. In comparison, the elevated SecoB concentrations measured in our post-exposure plasma samples were in the range of 0.589–1009.74 pmol/mL (nM). SecoB can induce endoplasmic reticulum stress in human umbilical vein endothelial cells at 1000nM and promote apoptosis at concentrations 5000nM [65], thus presenting a potential mechanistic link between O_3_-induced elevated circulating SecoB levels and cardiovascular dysfunction.

In contrast to SecoB, 27-hydroxycholesterol is enzymatically generated from cholesterol by CYP27A1 [68]. It is the most prevalent oxysterol in circulation in humans and highly prevalent in the lung compared to other organs [68]. Additionally, 27-hydroxycholesterol is an endogenous ligand for liver X receptor (LXR) that regulates cholesterol homeostasis [69]. In this role, 27-hydroxycholesterol and other oxysterols can be anti-inflammatory as activation of LXR results in transrepression of the NF-kB pathway [69–72]. Conversely, we have previously shown that SecoA, SecoB, β-epoxycholesterol, which are upregulated in the lung after O_3_ exposure and antagonize the LXR pathway and activate NF-kB signaling [55]. We observed a significant increase in 27-hydroxycholesterol in plasma from both asthmatic and non-asthmatic subjects and a decrease in sputum as well (Figs 2 and 3, S6 and S7 Tables). Exposure is not only associated with increases in concentrations of LXR antagonists, but also decrease in LXR agonists suggesting a potential net down regulation of LXR signaling in the lung. It is unclear what functional consequences stems from perturbations to oxysterol concentrations following ozone exposure. Therefore, future studies are needed to elucidate biological consequences associated with altered 27-hydroxycholesterol and other oxysterol concentrations in the lung and circulation. The LXR signaling pathway may serve as an integration site for altered metabolic cues from noxious environmental pollutants such as O_3_.

Prior to our study, transcriptomic studies conducted in rodents identified increased expression of cholesterol biosynthesis genes in the lung beginning 12–24 hours following O_3_ exposure [30–33] suggesting important roles for this pathway in O_3_ responses. In the sputum samples, we observed a significant decrease in several cholesterol precursors between asthmatics and non-asthmatics (Fig 2). These perturbations may require upregulation of cholesterol biosynthesis genes to return to a homeostatic state. Ultimately, the contribution of metabolic state and dyslipidemia to lung health following O_3_ exposure is not well understood. For this reason, we conducted an exploratory analysis using machine learning models to assess if profiles of sterols in plasma, were suitable predictors of O_3_ responder status. Our results indicated plasma sterols performed well for lung response prediction with cholesterol emerging as the top predictor (S11 Table). In addition, when cholesterol and 25-hydroxyvitamin D were chosen as the sole predictors for lung response, there was a clear separation between non-responders and responders that was highly associated with disease status (Fig 4). We continued analysis of association of plasma sterols with O_3_-induced changes in respiratory proinflammatory markers and lung function stratified by disease status (Fig 6). We found baseline plasma 25-hydroxyvitamin D levels to be positively correlated with post-exposure levels of uteroglobin and lung function measures, FEV1 and FVC. Conversely, baseline 25-hydroxyvitamin D levels were negatively correlated with post-exposure neutrophil percentages and MPO levels (Fig 6A), but these correlations were only present in non-asthmatics. Similar positive association between serum 25-hydroxyvitamin D levels and lung function measures, FEV1 and FVC, have been observed in the Center for Disease Control National Health and Nutrition Examination Survey [69]. Surprisingly, asthmatics had slightly lower 25-hydroxyvitamin D concentrations at baseline that increased following O_3_ exposure, potentially contributing to the lack of association (Fig 3B). Though none of our study participants were classified as vitamin D deficient (concentration of 25-hydroxyvitamin D < 50 nmol/L), the levels of vitamin D were significantly lower in black, male subjects in our study (S1 Data). This is consistent with a previous report that found, despite similar concentrations of vitamin D binding protein being across all racial groups, 25-hydroxyvitamin D levels were significantly lower in black subjects [70,71]. While current vitamin D recommendations are based on levels necessary for bone health, studies analyzing the association with additional disease outcomes, such as lung disease, may be needed in further refining these recommendations. Together, the data shown here suggest that circulating vitamin D levels above the recommended levels are associated with susceptibility to O_3_-induced inflammation and lung function changes.

While findings from our study may have broader clinical implications, it was exploratory in nature and is not without limitations. Notably, machine learning algorithms perform best when given a substantial amount of data, which helps train the model while keeping it generalizable to “unseen” data. Generating a large cohort size in clinical studies can be difficult, particularly in the current design with human volunteers undergoing controlled exposures to O_3_ followed by extensive molecular and phenotypic profiling pre- and post-exposure; however, it should be noted that machine learning methods can still inform underlying biology with similarly sized cohorts, as previously found [72,73]. In addition, the unequal distribution of inflammatory responders diminished the performance of the inflammatory response prediction for all models. Alternative strategies to address class imbalance by undersampling the majority class or oversampling the minority class have been previously employed [74,75]. Though we recognize that expanded study designs and replication cohorts would enhance the current study’s findings, our study is novel by employing machine learning to predict O_3_ responsiveness from oxysterol data, and all model performance parameters are openly reported. The asthmatics included in this study demonstrated muted lung function responses to O_3_ (S2 Table), though it is unclear whether this is a protective or maladaptive response to O_3_ [76]. Using a larger validation cohort would allow us to prospectively evaluate whether newly identified differences in sterols and factors such as sex, disease status, or vitamin D status, may modify susceptibility to O_3_-induced lung responses.

## Conclusion

We conducted this study to examine whether O_3_-induced changes in sterol and oxysterol profiles in both the lung and circulation of healthy and asthmatic individuals following O_3_ exposure associate with hallmarks of O_3_ responsiveness. We demonstrated that elevated concentrations of SecoB in plasma were associated with O_3_ exposure in both asthmatics and non-asthmatics. Considering that SecoB has been linked to cardiovascular disease, studies to understand if elevations in SecoB are associated with O_3_-induced cardiovascular outcomes long-term, are needed. In addition, our data highlight systemic metabolic factors, such as 25-hydroxyvitamin D and cholesterol, as potential correlates or predictors for pulmonary responses to O_3_. Additional studies in larger cohorts are needed to assess the impact of vitamin D status on environmental exposures. Surprisingly, the associations we observed are with vitamin D levels falling within the recommended range. Hence, studies designed to understand causative factors for the prevalence of vitamin D insufficiency and deficiency in populations susceptible to environmental lung diseases are critical.

## Supporting information

S1 Data Online data supplement. Figure S1: Workflow of sterol and oxysterol analysis from plasma and sputum samples. Figure S2: Baseline plasma 25-hydroxyvitamin D levels stratified by sex and race.
(DOCX)

S1 Table Subject demographics. (XLSX)

S2 Table Summary of lung function changes following ozone exposure. (XLSX)

S3 Table Induced sputum cell characteristics. (XLSX)

S4 Table Cytokine concentrations in induced sputum. (XLSX)

S5 Table Cytokine concentrations in plasma. (XLSX)

S6 Table Sterol and oxysterol concentrations in induced sputum. (XLSX)

S7 Table Sterol and oxysterol concentrations in plasma. (XLSX)

S8 Table Confusion matrix from support vector machine prediction of inflammatory or lung response status. (XLSX)

S9 Table Confusion matrix from classification prediction of inflammatory or lung response. (XLSX)

S10 Table Inflammatory response variable importance ranking. (XLSX)

S11 Table Lung response variable importance ranking. (XLSX)

## Abbreviations

O_3_: ozone
UPLC-MS: ultra high-performance liquid chromatrography mass spectrometry
Ppm: parts per million
ELISA: enzyme linked immunosorbent assay
MPO: myeloperoxidase
QRILC: quantile regression imputation of left-censored
FEV1: forced expiratory volume in one second
RF: random forest
SVM: support vector machine
KNN: k nearest neighbor
AUC: area under the curve
ROC: receiver operating characteristic
BMI: body mass index
FVC: forced vital capacity
SecoB: secosterol-B
Chol: cholesterol
Lan: lanosterol
dHLan: dihydrolanosterol
DHL– 24: dehydrolathosterol
14dZym: 14-dehydrozymosterol
Zym: zymosterol
8DHD: 8-dehydrodesmosterol
7DHD: 7- dehydrodesmosterol
Des: desmosterol
14dZyme: 14-dehydrozymostenol
Zyme: zymostenol
Lath: lathosterol
8-DHC: 8-dehydrocholesterol
7-DHC: 7-dehydrocholesterol
IL: interleukin
TARC: thymus and activation-regulated chemokine
bEP-Chol—b: epoxycholesterol
24OH-Chol: 24-hydroxycholesterol
25OH-Chol: 25-hydroxycholesterol
27OH-Chol: 27-hydroxycholesterol
ROS: reactive oxygen species
CYP: cytochrome p450
LXR: liver X receptor
NF-kB: nuclear factor kappa-light-chain-enhancer of activated B cells
