# Supplementary material for: Plasma sterols and vitamin D are correlates and predictors of ozone-induced inflammation in the lung: A pilot study
Source: PLoS One. 2023 May 15;18(5):e0285721. doi: 10.1371/journal.pone.0285721 (PMC10184915; doi:10.1371/journal.pone.0285721)
Supplement: S1 Data — Figure S1: Workflow of sterol and oxysterol analysis from plasma and sputum samples. Figure S2: Baseline plasma 25-hydroxyvitamin D levels stratified by sex and race. (DOCX) [file pone.0285721.s001.docx]

## **Online Data Supplement**

## Title: Plasma sterols and vitamin D are correlates and predictors of ozone-induced inflammation in the lung: A pilot study

Alexia N. Perryman^1,2^, Hye-Young H. Kim^3^, Alexis Payton^2,4^, Julia E. Rager^1,2,4^, Erin E. McNell^1^, Meghan E. Rebuli^1,2,5^, Heather Wells^2^, Martha Almond^2^, Jamie Antinori^2^, Neil E. Alexis^1,2,5^, Ned A. Porter^3^, Ilona Jaspers^1,2,4,5*^

^1^Curriculum in Toxicology and Environmental Medicine, University of North Carolina at Chapel Hill

^2^ Center for Environmental Medicine, Asthma, and Lung Biology, University of North Carolina at Chapel Hill

^3^ Department of Chemistry, Vanderbilt University

^4^Department of Environmental Sciences and Engineering, Gillings School of Global Public Health, University of North Carolina at Chapel Hill

^5^Department of Pediatrics, University of North Carolina at Chapel Hill

*Corresponding Author: Ilona Jaspers, email: [ilona_jaspers@med.unc.edu](mailto:ilona_jaspers@med.unc.edu)


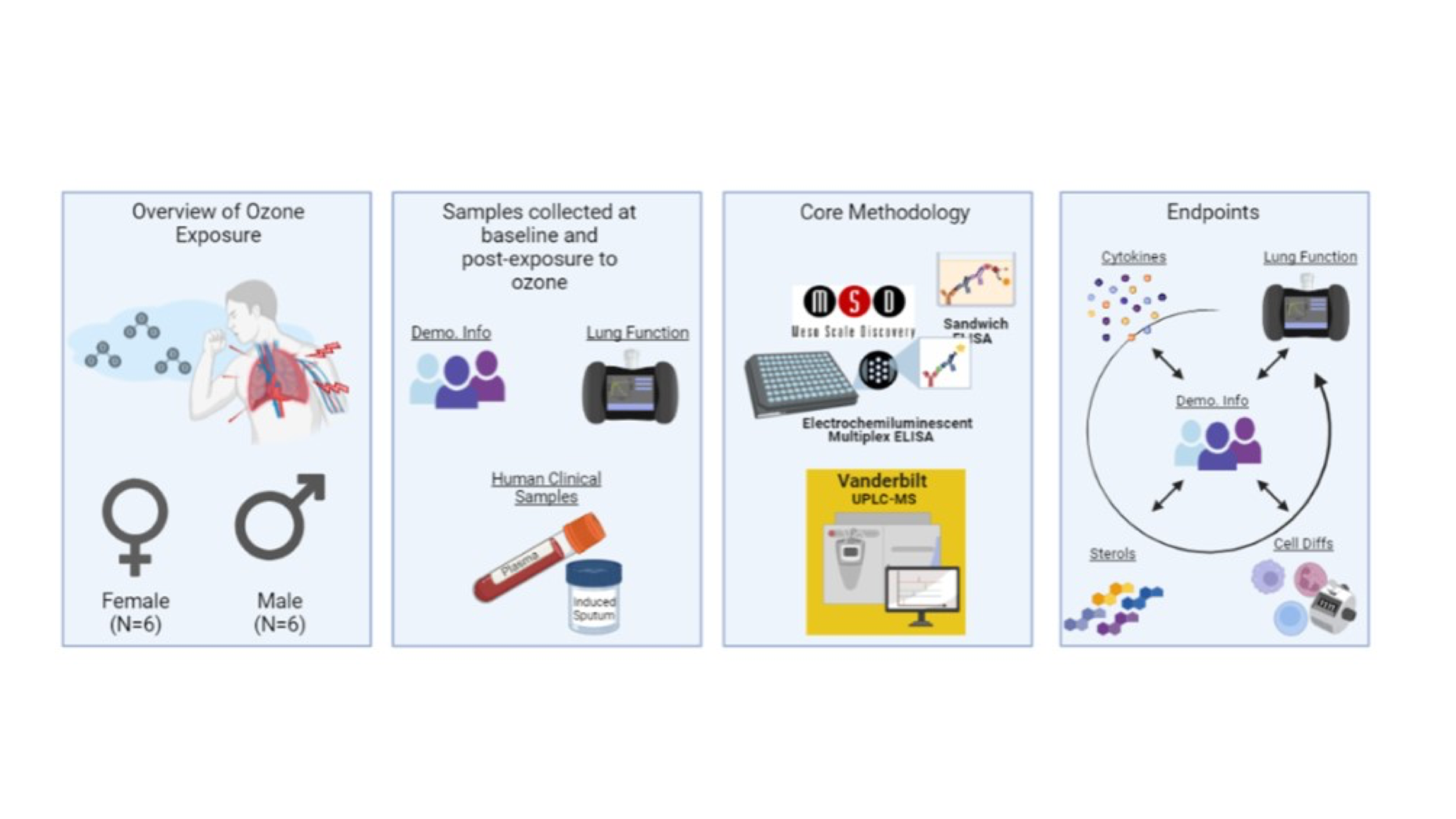
 Figure S1: Workflow of sterol and oxysterol analysis from plasma and sputum samples. Human volunteers were exposed to O_3_ (0.4ppm) for 2 hours. Collected plasma and sputum samples were analyzed for sterols and oxysterols by UPLC-MS. An additional 32 cytokines and soluble mediators were analyzed by singleplex and multiplex ELISA.

**Supplemental Methods**

Sterol and oxysterol profiling of plasma and sputum using N, N-dimethylglycine (DMG) derivatization and LC-MS analysis

For plasma and sputum, 100 μL or 400 μL were taken, respectively, and combined with 10 μL of antioxidant solution (2.5 mg triphenylphosphin and 1.0 mg butylated hydroxytoluene in 1mL ethanol) and 10 µL deuterated (d_6_/d_7_) internal standards (30 μM d_7_-Chol, d_6_-Lan, d_7_-dHLan and 3 μM d_7_-7-DHC, d_7_-8-DHC, d_6_-Des, d_6_-7-DHD, d_6_-8-DHD, d_7_-Lath, d_6_-DHL, d_7_-Zyme, d_6_-Zym, d_7_-14d-Zyme, d_6_-14d-Zym), 400 μL 0.9% NaCl, and 600 μL Folch solution (2:1 =chloroform: methanol, v:v). After vigorous vortex and centrifugation for 5 minutes at 5000g, the chloroform layer was collected, dried, and derivatized by addition of 100 μL of a fresh made N, N-dimethylglycine (DMG) solution (Per 1 mL of reagent: 20 mg 2-methyl-6-nitrobenzoic anhydride, 14 mg DMG, 6 mg 4-dimethylaminopyridine, 0.1 mL anhydrous triethylamine in 0.9 mL anhydrous chloroform). After 30 minutes of agitation at room temperature, the resulting solution was evaporated in Savant™ Speed Vacuum Concentrator and reconstituted in 100 μL of methanol. 10 μL was injected onto the column (Agilent Poroshell EC, 10 cm x 2.1 mm, 1.9 μm) using a solvent mixture of acetonitrile:methanol:water, 70:25:5 (0.01% (v) formic acid, 1 mM ammonium acetate) for 16 minutes runtime. A TSQ Quantum Ultra mass spectrometer (ThermoFisher) was used for MS detections, and data were acquired with a Finnigan Xcalibur (v. 2.2 SPI) software package. Selected reaction monitoring (SRM) of the DMG derivatives was acquired in the positive ion mode using electrospray ionization (ESI). All endogenous sterol intermediates and cholesterol metabolites were quantified by using the matching deuterated sterols. Response factors of each oxysterol SRM were measured relative to d_7_-cholesterol, and those response factors were used to quantify each oxysterol. Full list of SRM of sterols and oxysterols was reported previously (1). In addition, 504.31 🡪 355.30 for SecoA and 504.30 🡪 365.30 SecoB eluting at 2.0 and 2.7 minutes respectively were included in the analysis.

Quantification of Uteroglobin, Myeloperoxidase, and Proinflammatory Mediators

Proinflammatory mediators were quantified in plasma and induced sputum samples by electrochemiluminescent multiplex ELISA using the V-PLEX Human Cytokine 30-Plex Kit (MesoScale Discovery, Rockville, MD) for the following cytokines and chemokines: Eotaxin, Eotaxin-3, GM-CSF, IFN-γ, IL-1α, IL-1β, IL-2, IL-4, IL-5, IL-6, IL-7, IL-8, IL-10, IL-12p40, IL-12p70, IL-13, IL-15, IL-16, IL-17A, IP-10, MCP-1, MCP-4, MDC, MIP-1α, MIP-1β, TARC, TNF-α, TNF-β, and VEGF-A. Levels of myeloperoxidase were quantified in the induced sputum and plasma samples by single-plex ELISA (R&D Systems, Cat no: DY3174). Levels of uteroglobin (CCSP/CC16) were quantified in the induced sputum samples by single-plex ELISA (R&D Systems, Cat no: DY4218).

*Data Organization and Processing for Machine Learning*

When implementing background filters, variables with <25% of data within each sample type (i.e., plasma and sputum samples) and time point (i.e., pre or post O_3_ exposure) were removed. This resulted in the removal of the analyte, uteroglobin, in both pre and post O_3_ exposure in plasma samples. Subjects with <25% of data within each sample type, time point, and variable category (i.e., cell differential, cytokine, lung function, and sterol data). This resulted in the removal of the one male asthmatic subject from both cytokine and sterol sputum data prior to O_3_ exposure. Next, two different methods were used to perform data imputation. QRILC was used to impute missing data for cytokine, cell differential, and sterol measures because these missing values were likely attributable to low expression or missing not at random (MNAR), and QRILC imputes data from the left side of a normal distribution. Lung function imputation was performed using random forest since it generates missing data across the entirety a normal distribution since this data was missing at random (MAR) (3). Notably, data were imputed for each sample type, time point, and variable category, separately, to account for varying data distributions specific to each collected endpoint.

*Supervised Machine Learning Models to Predict Inflammatory and Lung Function Response*

Predictor variables evaluated in these models included sterol concentrations across plasma samples prior to O_3_ exposure since plasma samples are relatively less invasive and more universally acquirable than induced sputum.  Covariates including age, BMI, disease status, ethnicity, race, sex, and visit season were incorporated as additional predictors in adjusted models. Age and BMI were continuous, while all other covariates were categorical, including race which was dichotomized into White and non-White subjects. Visit season recorded the season in which the data was collected either Fall, Spring, Winter or Summer, because ambient O_3_ concentrations and vitamin D synthesis vary with the season.

Three supervised machine learning models were selected for evaluation, to build and test models that predict the binary classification of inflammatory and lung response: random forest (RF), support vector machine (SVM), and k nearest neighbor (KNN). RF constructs a multitude of decision trees and averages the results to ensure the best prediction. Each tree only uses a subset of predictors, allowing RF to decorrelate its predictors or features making this model more adaptable and compute faster (9). SVM utilizes a hyperplane that seeks to separate classes by maximizing the margin or the distance from each predictor to the hyperplane(10). On the other hand, KNN predicts based on the distance between a test point and all the training data, ranks the nearest neighbors (training points) based on that distance, and votes on the class of the k nearest neighbors (11).

Modeling was carried out using the following packages and associated parameters: RF models were run using the randomForest package in R (v4.6.14). These algorithms were tuned by changing the number of trees grown (including 50, 250, and 500 trees) and the number of random variables to be included in those trees (including the square root of the number of predictors (p), p/2, and p/3). The optimal model was selected if it had the lowest out-of-bag (OOB) or misclassification errors. In the instance that OOB errors were equal, parameters were then selected based on the largest number of trees grown, because the error rate plateaus at a certain point despite increasing the number of trees (9). If OOB errors were still equal between models, then the number of sampled predictors equaling sqrt(p) was selected as it is the default for classification RF models in the randomForest package. SVM models were run using the tune function from the e1071 package in R (v1.7.9.) (12).  The algorithm’s parameters were tuned by modifying the kernel, epsilon, cost, and gamma values. Linear, polynomial, and radial kernels were tested to form the hyperplane and decision boundaries in higher dimensions.  Epsilon defines the margin of tolerance for misclassification and was set to 0.035. The cost parameter is necessary for defining the penalty for misclassification and numbers were tested between 1-5. Gamma defines the amount of curvature of the hyperparameter (10). These values set between 0.1-0.2 were tried with the exception of polynomial kernels where the gamma was left as the default of 0.1. KNN models did not have tuned parameters, however the training set was centered and scaled prior to training the algorithm.

*Evaluation of Model Performance and Interpreting Best Performing Machine Learning Model*

Resulting models were compared to identify which model and set of predictor variables were best able to predict inflammatory and lung response. 5-fold cross-validation was implemented for all algorithms randomly splitting the data into training and testing sets to prevent models from overfitting to the training set. In 5-fold cross validation, subjects in the original dataset were divided into 5 equal sized groups or folds. Four of the folds were used to train and the last was used to test each model and this process was repeated five times (13). The final reported confusion matrix and AUC were averaged across the five cross validation iterations.

To determine the contribution of each predictor to RF model performance, variable importance ranks based on mean decrease gini were extracted (14). To our knowledge, these variable importance rankings are only able to be produced by RF. Additionally, RF models were rerun with five additional noise variables to further evaluate which of the original predictors were able to rank above the highest random noise variable and therefore not likely due to chance.  These noise variables were generated using the Sample function in base R to randomly permute values from the dataset.

**Supplemental Results**

Classical Hallmarks of O_3_ Responsiveness

O_3_ response in humans is clinically denoted by decrements in lung function, increase in markers of lung injury/permeability, and neutrophilic influx in the lung (1-3). All lung function measurements and sputum characteristics have been summarized and compared within disease status group by matched Wilcoxon sign rank test (Tables S2 and S3, respectively). In accordance with previously published findings from this cohort, we observed a significant decrease in lung function for FEV1 and FVC in non-asthmatics (Table S2). In contrast, we observed marginal decrease in FEV1 (p = 0.07) and a significant decrease in FVC in asthmatics (Table S2). An additional metric for classifying robust O_3_ responder status is a FEV1 decrement >5% (39, 40). Based on this designation, our study included 10 non-asthmatic responders (5 males/5 females), 2 asthmatic responders (1 male/1 female), 2 non-asthmatic non-responders (1 male/1 female) and 10 asthmatic non-responders (5 males/5 females).

We also observed a significant increase in sputum percent neutrophils and decrease in percent macrophages in accordance with prior published findings for both non-asthmatics and asthmatics (Table S3) (41).

*Confirmation of Baseline Differences in Asthmatic and Non-Asthmatic Study Participants*

We evaluated analytes for differences between asthmatics and non-asthmatics at baseline and post-exposure time points by mixed-effects model with repeated measures. The resulting p-values were adjusted for multiple comparisons using Benjamini-Hochberg correction and results were considered a discovery with q-value < 0.2. We observed several differences in cytokines consistent with macrophage polarization (15) and cytokine receptor γ-chain family cytokines at baseline (16) (Table S4 and S5). Asthmatics had higher concentrations of eotaxin-3 and MCP-4 and lower concentrations of IL-13 and IL-16 in sputum (Table S4). In plasma, asthmatics had higher concentrations of MDC, MCP-4, eotaxin, eotaxin-3, TARC, and IL-7 (Table S5). In contrast, asthmatics hadlower plasma IL-4, IL-13, and IL-15 concentrations (Table S5)

Additionally, we assessed whether cytokines in sputum and plasma samples from asthmatics and non-asthmatics differed at post-exposure time points independent of disease interaction with O_3_. At the post-exposure timepoint, the lower concentration of IL-13 and higher concentrations of eotaxin-3 and MCP-4 in sputum persisted (Table S4). Additionally, asthmatics had higher concentrations of IL-5, TARC, and lower concentrations of MCP-1, MIP-1β, IL-6, and uteroglobin in sputum (Table S4). In plasma, asthmatics still had higher concentrations of eotaxin-3, MDC, and MCP-4 (Table S5). Additionally, asthmatics had higher concentrations of IL-1α, IL-5, and MCP-1 in plasma (Table S5).

*O_3_ -associated Changes in Cytokines in Sputum and Plasma*

In sputum, we observed an increase in IL-6 for and significant decrease in IL-15 after O_3_ exposure for non-asthmatics only (Table S3). For asthmatic subjects, MCP-4 was significantly decreased in sputum after exposure.

In the plasma samples , we found IL-5 to be decreased in both asthmatics and non-asthmatics after O_3_ exposure (Table S5). There was a significant interaction between sex and O_3_ response for IL-16 and IL-5; however, it did not meet the cutoff following correctiion for multiple compairsons (Table S4=5). We observed a significant decrease in concentrations of IL-5, IL-8, IP-10, and MIP-1β in plasma from asthmatics after exposure (Table S3).

In addition to cytokines, we also quantified myeloperoxidase (MPO) and sputum uteroglobin/CC10/CC16/CCSP levels. Uteroglobin is one of the most robustly induced markers in response to O_3_ in plasma (17-19). Though increases in circulating uteroglobin are likely not specific to O_3_ and can reflect a broad range of lung insults, as reviewed previously, it has utility for identifying O_3_-induced lung injury (20). Unfortunately, we did not have sufficient sample volume or assay sensitivity to detect uteroglobin in our plasma samples. Neither MPO nor sputum uteroglobin concentrations were significantly different following O_3_ exposure (Table S4 and S5). However, after stratifying for sex, uteroglobin was significantly (interaction p = 0.0148) different in sexes in non-asthmatics (Table S4). Specifically, measured concentrations of sputum uteroglobin were not significantly different between males and females at baseline, but were significantly lower in males than in females following O_3_ exposure.

**SUPPLEMENTAL REFERENCES**

1. Tallman KA, Allen LB, Klingelsmith KB, Anderson A, Genaro-Mattos TC, Mirnics K, Porter NA, Korade Z. Prescription medications alter neuronal and glial cholesterol synthesis. *ACS chemical neuroscience* 2021;12(4):735-745.

2. Lazar C, Gatto L, Ferro M, Bruley C, Burger T. Accounting for the multiple natures of missing values in label-free quantitative proteomics data sets to compare imputation strategies. *J Proteome Res* 2016;15(4):1116-1125.

3. Stekhoven DJ, Bühlmann P. Missforest—non-parametric missing value imputation for mixed-type data. *Bioinformatics* 2011;28(1):112-118.

4. Hazucha MJ, Folinsbee LJ, Bromberg PA. Distribution and reproducibility of spirometric response to ozone by gender and age. *Journal of Applied Physiology* 2003;95(5):1917-1925.

5. Alexis NE, Lay JC, Haczku A, Gong H, Linn W, Hazucha MJ, Harris B, Tal-Singer R, Peden DB. Fluticasone propionate protects against ozone-induced airway inflammation and modified immune cell activation markers in healthy volunteers. *Environ Health Perspect* 2008;116(6):799-805.

6. Folinsbee LJ. Time course of response to ozone exposure in healthy adult females. *Inhal Toxicol*;12(3):151-167.

7. Clark J, Avula V, Ring C, Eaves LA, Howard T, Santos HP, Smeester L, Bangma JT, O'Shea TM, Fry RC, et al. Comparing the predictivity of human placental gene, microrna, and cpg methylation signatures in relation to perinatal outcomes. *Toxicol Sci* 2021;183(2):269-284.

8. Ring C, Sipes NS, Hsieh JH, Carberry C, Koval LE, Klaren WD, Harris MA, Auerbach SS, Rager JE. Predictive modeling of biological responses in the rat liver using in vitro tox21 bioactivity: Benefits from high-throughput toxicokinetics. *Computational toxicology (Amsterdam, Netherlands)* 2021;18.

9. Breiman L. Random forests. *Machine Learning* 2001;45(1):5-32.

10. Noble WS. What is a support vector machine? *Nature Biotechnology* 2006;24(12):1565-1567.

11. Batista GEAPA, Silva DF. How k-nearest neighbor parameters affect its performance.2009.

12. Meyer D, Dimitriadou E, Hornik K, Weingessel A, Leisch F. Misc functions of the department of statistics, probabilitytheory group (formerly: E1071), tu wien.2015.

13. Berrar D. Cross-validation.2018.

14. Martinez-Taboada F, Redondo JI. The siesta (seaav integrated evaluation sedation tool for anaesthesia) project: Initial development of a multifactorial sedation assessment tool for dogs. *PLOS ONE* 2020;15(4):e0230799.

15. Chung KF, Barnes PJ. Cytokines in asthma. *Thorax* 1999;54(9):825.

16. Rochman Y, Spolski R, Leonard WJ. New insights into the regulation of t cells by γc family cytokines. *Nature Reviews Immunology* 2009;9(7):480-490.

17. Blomberg A, Mudway I, Svensson M, Hagenbjörk-Gustafsson A, Thomasson L, Helleday R, Dumont X, Forsberg B, Nordberg G, Bernard A. Clara cell protein as a biomarker for ozone-induced lung injury in humans. *European Respiratory Journal* 2003;22(6):883-888.

18. Arjomandi M, Balmes JR, Frampton MW, Bromberg P, Rich DQ, Stark P, Alexis NE, Costantini M, Hollenbeck-Pringle D, Dagincourt N, et al. Respiratory responses to ozone exposure. Moses (the multicenter ozone study in older subjects). *Am J Respir Crit Care Med* 2018;197(10):1319-1327.

19. Broeckaert F, Arsalane K, Hermans C, Bergamaschi E, Brustolin A, Mutti A, Bernard A. Serum clara cell protein: A sensitive biomarker of increased lung epithelium permeability caused by ambient ozone. *Environmental Health Perspectives* 2000;108(6):533-537.

20. Lakind JS, Holgate ST, Ownby DR, Mansur AH, Helms PJ, Pyatt D, Hays SM. A critical review of the use of clara cell secretory protein (cc16) as a biomarker of acute or chronic pulmonary effects. *Biomarkers* 2007;12(5):445-467.

###### Figure S2: Baseline plasma 25-hydroxyvitamin D levels stratified by sex and race. 25-hydroxyvitamin D (25OH-VitD)levels were quantified in plasma samples. Cut-off values for vitamin D insufficiency (green, <50 pmol/mL) and vitamin D deficiency (red, <25 pmol/mL) are overlaid. Groups were compared by unpaired Wilcoxon signed rank test. Error bars represent mean ± SEM. *p<0.05, **p<0.01. All (N=12), Female (N=6),Male (N=6), Non-black (N=9), Black (N=3)
